# Increasing prehospital tourniquet use attributed to non-indicated use: an 11-year retrospective study

**DOI:** 10.1007/s00068-024-02716-3

**Published:** 2025-01-24

**Authors:** Daniel J. Hedger, Mitchell Smith, Natasha Weaver, Jason Bendall, Zsolt J. Balogh

**Affiliations:** 1 Department of Traumatology, John Hunter Hospital, Newcastle, NSW 2310 Australia; 2 Discipline of Surgery, School of Medicine and Public Health, University of Newcastle, Newcastle, NSW Australia; 3 Department of Anaesthesia, John Hunter Hospital, Newcastle, NSW 2310 Australia; 4 Department of Traumatology, Division of Surgery, John Hunter Hospital and University of Newcastle, Locked Bag 1, Hunter Region Mail Centre, Newcastle, NSW 2310 Australia

**Keywords:** Prehospital tourniquet, Haemorrhage control, Civilian trauma

## Abstract

**Purpose:**

The use of prehospital tourniquets (PHTQ) for haemorrhage control in the civilian trauma population has increased over the past decade with some reports documenting the overuse of the device. The aim of this study was to identify the proportion of PHTQ use that is non-indicated and determine how this proportion is changing over time.

**Methods:**

An 11-year retrospective study was performed at a Level-1 Trauma Centre on all trauma patients admitted with a PHTQ. Local PHTQ guidelines were used to define non-indicated use. Collected variables included patient demographics, injury characteristics, tourniquet application characteristics, prehospital data, emergency department data, and clinical outcomes. The primary outcome was non-indicated PHTQ use. The secondary outcome was complications attributed to PHTQ use.

**Results:**

There were 88 PHTQ applications to 88 extremity injuries in 86 patients (*n* = 86, median (IQR) age 43 (28–57) years, 85% male). PHTQ use was deemed non-indicated in 68 cases (68/88, 77% [95%CI 67-86%]). The proportion of non-indicated PHTQ use increased over the period of the study period (*p = 0.03*). At least one complication potentially from PHTQ use was seen in 33 patients (33/86, 38%). In patients with prolonged tourniquet time (*n* = 13), at least one complication from PHTQ use was seen in 11 patients (11/13, 85%).

**Conclusion:**

Over this 11-year period, we identified that the increase in PHTQ use in civilian trauma is from increasing non-indicated use. Given that complications are associated with unnecessary PHTQ use, the adherence to the guidelines needs to be urgently reinforced.

**Supplementary Information:**

The online version contains supplementary material available at 10.1007/s00068-024-02716-3.

## Background

The use of prehospital tourniquets (PHTQ) for haemorrhage control in civilian populations has seen a widespread and rapid increase over the past decade since their introduction in prehospital protocols [1, 2, 3]. Whilst PHTQ use had traditionally been controversial, their increased use in military populations demonstrated that they could be used safely and effectively for haemorrhage control on the battlefield [4, 5, 6]. There remains conflicting perspectives regarding the applicability of military data to civilian trauma, due to differences in baseline population, indications for tourniquet use, injury mechanism, and the absence of the challenging battlefield environment [7, 8, 9].

The increased use of PHTQ in the civilian setting has been accelerated by the increasing incidence of mass-casualty events world-wide [9, 10, 11]. Educational campaigns that were developed in response to these events such as the “Stop the Bleed” campaign, initiated by the American College of Surgeons, were instrumental in educating civilian and prehospital medical staff in the use of PHTQ for haemorrhage control [11].

Concerns remain that the increased use of PHTQ has occurred in the absence of high-quality evidence in civilian populations [9, 10, 11, 12]. Despite this, it is clear that PHTQs are a necessary and lifesaving intervention in the rare cases where extremity haemorrhage cannot be controlled by other safe methods [13, 14, 15]. For this reason, their use has been emphasised and prioritised in clinical practice guidelines [16, 17]. These recommendations have largely been based on studies which have reported safety (i.e. benefits outweigh risks) and shown their use can reduce mortality and morbidity associated with extremity arterial injury [18, 19, 20, 21].

Whilst PHTQs can be lifesaving interventions when indicated, concerns exist that the rapid increase in PHTQ use is a result of increased use in patients who would be unlikely to benefit from their use, such as patients with isolated venous injuries or open fractures, where potentially other less restrictive means of haemorrhage control may have been effective. Some reports have shown that up to 50% of PHTQ use is in cases that would be considered non-indicated by current guidelines [16, 17, 22, 23, 24].

The aim of this study was to determine what proportion of PHTQ use is non-indicated and how this proportion is changing over time. We hypothesised that increasing PHTQ use is a result of increased non-indicated use as defined by local pre-hospital guidelines.

## Methods

### Study setting

This retrospective, observational cohort study was conducted at a university-affiliated level-1 tertiary trauma centre which admits over 4500 trauma patients annually, 10% of which have an injury severity score (ISS) of more than 15 [25].

### Ethical consideration

#### Ethics approval

was obtained from local clinical governance (AU202211-13).

### Study design

All patients were identified from the clinical records of the state-wide ambulance service (emergency medical service) crewed by registered paramedics and helicopter retrieval teams (doctor-paramedic) who transported patients to our trauma centre. Records were searched to identify cases in which a tourniquet had been applied in the prehospital setting between 01/01/2011 and 31/12/2021. Cases were then matched manually to patient electronic medical records (eMR) and to the institutional trauma registry. Patient eMR was then reviewed to determine if patients fulfilled the inclusion and exclusion criteria. Inclusion criteria included any patient who arrived at our institution with a PHTQ applied to an extremity for the purpose of haemorrhage control. Exclusion criteria included patients with a PHTQ applied for reasons other than haemorrhage control (e.g. crushed limb extractions), and non-traumatic haemorrhage control (e.g. arteriovenous-fistula bleeds). The Strengthening the Reporting of Observational Studies in Epidemiology (STROBE) statement guideline was used to ensure proper reporting of methods, results, and discussion (Supplemental Digital Content, Supplementary Data 1).

### Prehospital guidelines

At the time of this study the primary prehospital indication for applying an arterial tourniquet was extreme life-threatening arterial limb haemorrhage due to penetrating trauma or limb amputation not controlled by direct pressure [26], consistent with local resuscitation guidelines [17]. Other indications include when haemorrhage area is inaccessible and life-threatening arterial haemorrhage suspected. (e.g. entrapment) and mass / multiple casualties with life threatening arterial haemorrhage in triage mode where direct pressure cannot be provided without compromising other patients [26]. These guidelines emphasise that indications for tourniquet use will be rare in the pre-hospital setting and that the majority of external haemorrhage can be controlled using a graduated approach to haemorrhage control (direct pressure -> elevation -> tourniquet) [26]. These guidelines were first implemented in 2011 and did not change over the study period [26]. These guidelines are region specific, and there is currently no agreed upon definition of indication for PHTQ use [9]. Local prehospital services (both state-wide ambulance service and helicopter retrieval teams) used both the *Mechanical Advantage Tourniquet* (MAT^®^ - PYNG Medical, Richmond, British Columbia, Canada) and *Special Operations Force Tactical Tourniquet-Wide* (SOF^®^TT-W - TacMed, Coffs Harbour, NSW, Australia) tourniquets over the study period. Both tourniquets are non-pneumatic, mechanical strap style tourniquets.

### Collected variables

Variables collected included patient demographics (age, sex, year of injury, AIS extremity, ISS, comorbidities), injury characteristics (injury type, mechanism, intoxication), tourniquet characteristics (prior attempts at haemorrhage control, type of tourniquet applied, person who applied the tourniquet, which limb the tourniquet was applied to, time of injury to tourniquet placement, total tourniquet time, effectiveness of tourniquet), prehospital interventions and disposition (estimated blood loss, intubation, vital signs, blood transfusion, intravenous fluids, scene time), emergency department (ED) interventions and disposition (vital signs, laboratory results, massive transfusion protocol, ED interventions, success of haemostasis), and outcomes (presence of arterial injury, site of arterial injury, characteristics of arterial injury, interventions, complications, total units of blood transfused, intensive care unit (ICU) admission, length of hospital stay, mortality).

### Outcomes

The primary outcome for this study was the presence of non-indicated tourniquet use. For the purpose of this study, non-indicated tourniquet use was defined as tourniquets being applied in the absence of a potentially haemorrhaging arterial injury (confirmed by imaging or intraoperative findings) or being applied to arterial injuries where haemostasis likely could have been achieved without a tourniquet (determined by haemostasis being maintained in the ED following tourniquet removal using local non-surgical methods including direct pressure, limb positioning, and haemostatic gauze). Determination of the primary outcome was made by the primary author based on these criteria, with any questionable cases discussed with the senior author and treating clinicians. The secondary outcome for this study was complications attributed to tourniquet use. The following complications relevant to tourniquet use were collected: nerve palsy, acute kidney injury, rhabdomyolysis, wound infection, deep vein thrombosis (DVT)/venous thromboembolism (VTE), and amputation. These complications were selected based on a review of the current literature of PHTQ use [8, 12, 27].

### Data analysis

Demographic information, injury characteristics, and clinical variables were summarised for the sample. Continuous variables were summarised as median value with the interquartile range (Q1, Q3). Categorical data are summarised as frequency counts with percentages. The primary outcome was reported as a proportion with its exact binomial confidence interval. A Chi-squared test for trend was performed to determine significance of change in proportion of indicated vs. non-indicated tourniquet use over the 11-year study period.

Clinical complications and outcomes were summarised for the total sample and for the subgroups of patients without arterial injury and patients with prolonged tourniquet use. For the purpose of this study, prolonged tourniquet time was defined as total tourniquet time more than 120 min. No hypothesis tests comparing these subgroups were performed due to small group sizes. Data analysis was performed in Stata 16 (StataCorp, College Station, TX, USA).

## Results

### Demographics and injury characteristics

**Fig. 1:**
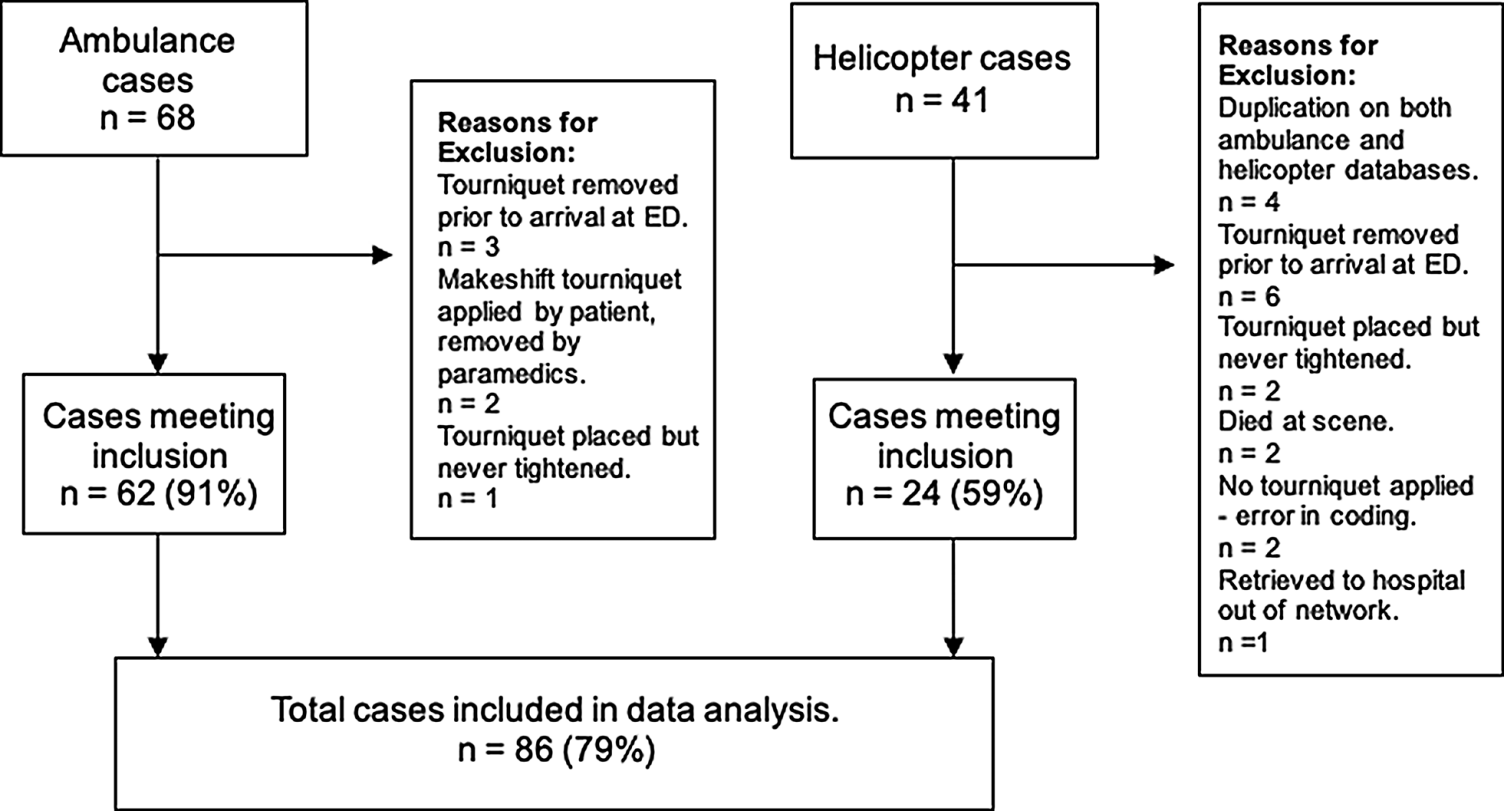
Flowchart showing patient inclusion and reasons for exclusion

**Fig. 2:**
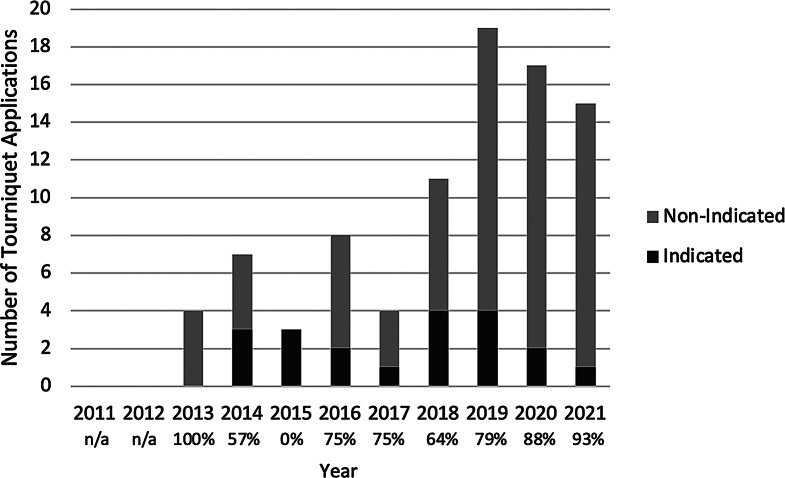
Frequency of indicated and non-indicated tourniquet use over the study period. Percentage of non-indicated/indicated is shown with each year

Demographic data are presented in Table 1. One-hundred and nine patients were identified from ambulance (68 patients) and helicopter (41 patients) records. Twenty-three (23/109, 21%) patients were excluded due to not meeting inclusion criteria. Reasons for exclusion are shown on Fig. 1. Eighty-six patients were included in full data analysis. The number of cases each year is shown on Fig. 2. Twenty-six patients (26/86, 30%) had an ISS > 15. The breakdown of injury mechanism is shown in Table 2. Seventy-two patients (72/86, 84%) had an isolated extremity injury to which the tourniquet was applied, whilst the remaining patients had the tourniquet applied to an extremity injury in the context of polytrauma.

**Table 1:** Patient clinical and demographic characteristics

Age: median (IQR), years	43 (28, 57)
Male gender, n (%)	73 (85%)
AIS extremity, median (IQR)	3 (1, 3)
ISS, median (IQR)	10 (2, 16)
**Comorbidities**,** n (%)**	
Hypertension	14 (16%)
Type 1 Diabetes Mellitus	1 (1.2%)
Type 2 Diabetes Mellitus	9 (11%)
Asthma	6 (7.0%)
COPD	2 (2.3%)
Anaemia	1 (1.2%)
Obesity	2 (2.3%)
Congestive Heart Failure	1 (1.2%)
Ischaemic Heart Disease	6 (7.0%)
Dyslipidaemia	7 (8.1%)
Epilepsy	3 (3.5%)
Mental Health Disorder	26 (30%)
Active Smoker	27 (31%)
EtOH use disorder	23 (27%)
Substance use disorder (other than EtOH)	16 (19%)
Hepatitis C	2 (2.3%)
Stroke	1 (1.2%)
Anticoagulant therapy	1 (1.2%)
No Comorbidities Documented	28 (33%)
**Intoxication**,** n (%)**	
Any	28 (33%)
EtOH	25 (29%)
Cannabis	4 (4.7%)
Amphetamines	7 (8.1%)
Opioids	2 (2.3%)
**Prehospital Vital parameters**	
SBP: median (IQR), mmHg	115 (90, 132)
HR: median (IQR), bpm	98 (83, 110)
GCS, median (IQR)	15 (14, 15)
Shock Index, median (IQR)	0.78 (0.68, 1.02)
**ED Vital parameters**	
SBP: median (IQR), mmHg	118 (102, 131)
HR: median (IQR), bpm	95 (80, 110)
GCS, median (IQR)	15 (14, 15)
Shock Index, median (IQR)	0.78 (0.65, 0.96)
**Admission laboratory parameters**	
pH, median (IQR)	7.33 (7.26, 7.40)
Lactate: median (IQR), mmol/L	2.8 (2.0, 3.78)
BD: median (IQR), mEq/L	-2.7 (-4.4, -0.3)
Hb: median (IQR), g/L	125 (111, 139)
INR, median (IQR)	1.1 (0.9, 1.3)

Continuous data are presented as medians with inter-quartile range (IQR). Categorical data are presented as frequency counts and percentages

**Table 2:** Mechanism of injury for 86 patients with PHTQ applied

Mechanism of injury	*n* (%)
**Penetrating**	60 (70%)
Accidental Laceration	26 (30%)
Self-Inflicted Laceration	10 (12%)
Home power-tool laceration	9 (11%)
Workplace / Industrial	6 (7.0%)
Assault / stabbing	5 (5.9%)
Impaled / Penetrating	2 (2.3%)
Accidental crossbow shooting	1 (1.2%)
Animal bite	1 (1.2%)
**Blunt / Crush**	**20 (23%)**
Motor vehicle collision (MVC)	4 (4.7%)
Motor Bike Collision (MBC)	8 (9.4%)
MV vs. Pedestrian	4 (4.7%)
Workplace / Industrial	2 (2.3%)
Fall	1 (1.2%)
Arterial aneurysm trauma	1 (1.2%)
**Traumatic Amputation**	**5 (5.8%)**
Motor vehicle collision (MVC)	1 (1.2%)
Motor Bike Collision (MBC)	1 (1.2%)
Assault / Machete	1 (1.2%)
Workplace / Industrial	2 (2.4%)
**Blast trauma**	**1 (1.2%)**
Workplace / Industrial Explosion	1 (1.2%)

Categorical data are presented as frequency counts and percentages

### Tourniquet

Tourniquet application data are presented in Table 3. There were 88 tourniquet applications to 88 extremities in 86 patients. In two patients (2/86, 2.3%), two tourniquets were applied to two different extremity injuries. Fourteen cases (14/88, 16%) had prolonged tourniquet time (> 120 min). Tourniquet was not effective (as defined as having pulses present or ongoing bleeding) in 12 cases (12/88, 14%).

**Table 3:** Tourniquet application data for 88 tourniquet applications to 88 limbs

**Tourniquet application**	
No documented attempt on haemorrhage control prior to PHTQ placement, n (%)	58 (66%)
Improvised tourniquet placed by civilian prior to placement of commercial PHTQ, n (%)	9 (10%)
Time from injury to PHTQ placement: median (IQR), min	25 (16, 38)
Total PHTQ time: median (IQR) min	70 (47, 103)
Time from arrival in ED to PHTQ removal: median (IQR), min	19.5 (7.25, 34)
PHTQ removed in operating room, n (%)	14 (16%)
Time from arrival in ED to removal in operating room: median (IQR), min	50 (31, 106)
**Person who applied tourniquet**,** n (%)**	
Paramedic staff	68 (77%)
Prehospital doctor	19 (22%)
Police	1 (1.1%)
**Distribution of limb application**,** n (%)**	
Left arm	24 (27%)
Right arm	27 (31%)
Left leg	17 (19%)
Right leg	20 (23%)
**Prehospital times**,** median (IQR)**,** min**	
Scene time	23 (17, 34)
Transport time	39 (29, 59)
Total prehospital time	62 (47, 97)

Continuous data are presented as medians with inter-quartile range (IQR). Categorical data are presented as frequency counts and percentages

### Prehospital interventions

Prehospital vital parameters are presented in Table 1. Sixty-six patients (66/86, 77%) had a shock index < 1. Thirteen patients (13/86, 15%) were intubated at the scene. Estimated blood loss was only recorded for 41 patients (41/86, 48%). Median blood loss for these cases was 1000 mL (350, 1000). Thirteen patients (13/86, 15%) received blood transfusion prior to arrival in ED. Median volume of fluid transfused prior to arrival in ED was 150 mL (0, 500).

### Emergency department interventions and disposition

ED vital parameters and laboratory parameters are shown in Table 1. Sixty-six patients (66/86, 77%) of patients had a shock index < 1. Massive transfusion protocols were activated in 21 patients (21/86, 24%). Most patients (51/86, 59%) did not receive blood transfusion. Computed tomography angiography (CTA) was performed in 29 patients (29/86, 34%). Three patients (3/86, 3.5%) received no interventions in ED and went straight to the operating room. Seven patients (7/86, 8.1%) had wound irrigation and closure in ED and were discharged without admission.

### Primary outcome

Tourniquet use was deemed non-indicated in 68 cases (68/88, 77%, 95%CI 67-86%). There was a statistically significant increase in the proportion of non-indicated to indicated tourniquet use during this period (*p = 0.03*). Forty-three extremities (43/88, 49%) did not have a potentially haemorrhaging arterial Injury. In cases with a potentially haemorrhaging arterial injury (44/88, 50%), haemostasis was able to be maintained after tourniquet removal using local non-surgical methods in 25 cases (25/44, 57%). In 20 of these cases (20/25, 80%), haemostasis was achieved with direct pressure and/or limb positioning alone. In five cases (5/25, 20%) haemostatic dressings were also used in conjunction with direct pressure and limb positioning.

Among the forty-four extremities (44/88, 50%) with confirmed arterial injury, four (4/44, 9.1%) had two arterial injuries. Presence of arterial injury was confirmed in operating room by arterial repair in 38 cases (38/48, 79%), and arterial ligation in 10 cases (10/48, 21%). CTA confirmed the arterial injury in 6 cases (6/48, 13%) prior to operative management. One patient who had a tourniquet applied to two different extremity injuries had confirmed arterial injury in one limb but not the other. In one case with open fracture, arterial injury on the extremity to which the tourniquet was applied was unable to be determined as the patient died in ED from other injuries prior to investigation following prehospital arrest from bilateral pneumothoraces.

Upper limb arterial injuries accounted for 35 cases (35/48, 73%). The most commonly injured arteries were the radial artery (16/48, 33%) and the ulna artery (13/48, 27%). The most commonly injured lower limb arteries were the popliteal artery (4/48, 8.3%) and the posterior tibial artery (4/48, 8.3%). Thirty-three arterial injuries (33/48, 69%) were classified as complete injuries, 10 (10/48, 21%) were classified as partial injuries, and four (4/48, 8.3%) were not classified in the operation report.

The most common injury in extremities without arterial injury was an isolated venous injury, accounting for 28 cases (28/43, 65%). Open fractures were the second most common, accounting for 12 cases (12/43, 28%). The remaining cases included two degloving injuries (2/43, 4.7%) and one case of a mangled extremity (1/43, 2.3%).

### Interventions

Seventy-seven patients (77/86, 90%) required intervention in the operating room. Eighteen patients (18/86, 21%) required only exploration, irrigation, and debridement. Thirty-nine patients (39/86, 45%) required some form of arterial vascular intervention (ligation, repair, or shunt placement). Thirty patients required nerve repair (30/86, 35%). Nine patients (9/86, 10%) underwent amputation. Three patients (3/86, 3.5%) had replantation of a limb. Median number of operative interventions was 1 (1, 2). Seven patients (7/86, 8.1%) had unplanned return to operating room.

### Complications

Complication rates are shown in Table 4. Thirty-three patients (33/86, 38%) had at least one recorded complication which may be attributed to tourniquet use. Among the 16 patients (16/86, 19%) with nerve palsy, 11 (11/16, 69%) had a nerve injury secondary to the primary injury documented on the operation report. Among the four cases (4/86, 4.7%) of deep vein thrombosis, two occurred in the extremity the tourniquet was applied to, and two were pulmonary embolisms without a confirmed primary location.

**Table 4:** Complication rates

Complication	Total patients *n* = 86	Patients without arterial injury *n* = 42	Prolonged tourniquet time *n* = 13	Median tourniquet time: median (IQR), min
No complication, n (%)	53 (62%)	28 (67%)	2 (15%)	60 (40,89)
Nerve Palsy, n (%)	16 (19%)	4 (9.5%)	4 (31%)	79 (60, 115)
AKI, n (%)	5 (5.8%)	3 (7.1%)	2 (15%)	81 (50, 240)
Rhabdomyolysis, n (%)	10 (12%)	6 (14%)	3 (23%)	103 (81, 120)
Wound Infection, n (%)	13 (15%)	8 (19%)	6 (46%)	116 (88, 195)
DVT / VTE, n (%)	4 (4.7%)	1 (2.4%)	2 (15%)	133 (84, 176)
Amputation, n (%)	9 (10%)	4 (9.5%)	5 (38%)	174 (113, 229)

Median tourniquet time is presented as medians with inter-quartile range (IQR). Categorical data are presented as frequency counts and percentages. Table is ordered by increasing median tourniquet time

Among the nine patients (9/86, 10%) that underwent amputation in operating room, two were formalisation of partial traumatic amputations (2/9, 22%), three were amputated secondary to unsalvageable limb with associated arterial injury (3/9, 33%), and four were amputated without any evidence of arterial injury due to the extensive tissue loss unlikely to lead to meaningful functional outcome (4/9, 44%). Among the patients who required amputation without arterial injury, median tourniquet time was 160 min (121, 191).

### Subgroup analysis

Two post-hoc subgroup analyses were performed for patients without arterial injury and for patients who had extended tourniquet time (Table 4). For cases without arterial injury, 30 (30/43, 70%) had no documented attempt at haemorrhage control prior to tourniquet placement. Six cases (6/43, 14%) that did not have arterial injury had extended tourniquet time, with median total tourniquet time 145 min (126, 174). Fourteen patients (14/42, 33%) who had no arterial injury had a recorded complication. Eleven patients (11/13, 85%) with extended tourniquet time had a recorded complication.

### Clinical outcomes

Clinical outcomes are presented in Table 4. Three patients died (3/86, 3.5%), two of whom died prior to intervention in operating room from their injuries (one in cardiac arrest on arrival and one who went into cardiac arrest in ED), and one who died after intervention on day two of admission due to multiple organ failure secondary to traumatic shock. Twenty-three patients (23/86, 27%) required ICU admission with a median ICU stay of two (1,7) days. Median length of hospital stay was 3 (1, 16) days. Length of hospital stay was 1 day or less in 27 patients (27/86, 31%). Thirty-nine patients (39/86, 45%) required blood transfusions during admission. Sixty-six patients (66/86, 77%) attended planned follow-up clinics. Two patients (2/86, 2.3%) discharged against medical advice.

## Discussion

The primary purpose of this retrospective review was to identify the proportion of PHTQ use that is non-indicated and determine how this proportion has changed over time. Our results confirm that a large proportion of PHTQ use is non-indicated and that increasing incidence of PHTQ use in our study is the result of increasing non-indicated use which may have been prevented by following current pre-hospital guidelines.

Our data confirms that there has been a significant increase in the use of pre-hospital tourniquet use in Australia since their introduction to prehospital protocols. This demonstrates that the wide-spread and rapid increase in tourniquet use in the United States [1, 2, 3] is also occurring in Australia. A key finding of this study was that whilst the use of PHTQ is increasing, this increase can be attributed to increasing non-indicated use. The number of indicated episodes of use remained relatively constant throughout the study period (Fig. 2). Whilst the relative numbers of PHTQ use have seen substantial increases, it is important to emphasise that it is still an uncommon intervention relative to the total number of trauma admissions seen at our trauma service.

This study showed that half of the patients who had PHTQ applied for extremity haemorrhage did not have an arterial injury. International data suggests that this is not an isolated finding, and the use of PHTQs in patients without arterial injuries is a common occurrence [9, 22, 23, 24, 27].

A key finding of this study was that 66% of total cases, and 70% of cases without arterial injury, had no documented attempt at haemorrhage control prior to PHTQ placement. These findings are especially significant as we found that even in patients with arterial injury, haemostasis could be maintained in the ED in 57% of cases after tourniquet removal using other safe methods of haemorrhage control. This highlights the fact that even when arterial haemorrhage is present, a graduated approach to haemorrhage control should still be applied as per local pre-hospital guidelines and current resuscitation guidelines [17, 26]. Furthermore, the radial and ulnar arteries were the most frequently injured arteries, arteries which are typically compressible and should be amenable to a graduated approach to haemorrhage control. These instances of PHTQ use may have been prevented had a graduated approach to haemorrhage control been used. This would have reduced the number of patients unlikely to have benefited from PHTQ without exposing them to potential harm.

Non-indicated tourniquet use is not without harm [22, 23]. Whilst it is difficult to retrospectively attribute complications to the tourniquet use, the initial trauma, or caused by surgical intervention, a previous study has demonstrated patients developing complications as a direct consequence of tourniquet use that was not clinically indicated [22]. In the current study, 33% of patients who had a tourniquet applied in the absence of an arterial injury developed a complication which could be secondary to tourniquet use. Furthermore, the subgroup analysis of patients with prolonged tourniquet use had higher rates of complications, suggesting that some of the complications observed could have been secondary to the tourniquets themselves. Interestingly, 75% of the patients who required limb amputation in the absence of arterial injury had prolonged tourniquet time. This supports a prior study which showed an increase in amputation rates in limbs without arterial injury which had PHTQs applied [23]. The authors acknowledge that attribution of complications to tourniquet use is difficult and that primary aim of this study was to assess whether tourniquet use was indicated and not assess for complications. Future prospective studies are required to further investigate the potential harms of non-indicated tourniquet use.

Whilst prolonged tourniquet use was only identified in 14 cases of tourniquet application, it is important to note that six of these cases did not have a potentially haemorrhaging arterial injury. In these cases, the use of a prolonged-tourniquet and its associated morbidity could have been preventable. Prolonged tourniquet use is a potential area where current guidelines could be improved, as current local and international guidelines recommend that tourniquets should remain in place until arrival at definitive care [17, 24, 26]. Our initial data search identified nine cases of PHTQ use which were excluded due to removal of the tourniquet prior to arrival in ED. The majority of these were cases where prehospital doctors had applied further haemorrhage control measures after tourniquet placement such as elevation, compression bandages, and haemostatic bandages, and then reassessed the need for tourniquet use. The reassessment of tourniquet use is especially pertinent with the extended tourniquet times that can be seen with retrieval of rural and remote patients. This supports recent calls for all providers trained in the application of PHTQ use to also be trained in the safe conversion or replacement of a PHTQ prior to arrival at definitive care [24].

The reassessment of tourniquets after application is also important to identify ineffective tourniquet use. Ineffective tourniquet use was found in 14% of cases. This finding is in line with an earlier study which demonstrated 10% of patients had ineffective tourniquets that were also associated with increased morbidity [22]. Ineffective tourniquet use increases the risk of complications and worsens blood loss by blocking venous return [28, 29].

There was a higher incidence of non-indicated tourniquet use in lower-limb injuries. The disproportionate application of tourniquets to lower limb injuries could be explained by the higher rates of open fracture in lower limbs. Analysis of injuries without arterial injury reveals that 33% were applied to open fractures, 79% of which occurred in the lower limb. Similar findings in international data suggests that a high proportion of PHTQs are being applied to open fractures [27]. Open fractures are not an indication for PHTQ use in local guidelines [26].

The finding that 67% of patients had at least one documented co-morbidity is noteworthy as it underscores the potential differences between civilian and military populations in terms of baseline health. PHTQ use in patients with diabetes carries a significantly higher risk of acute kidney injury than previously reported in military settings [7]. 12% of our study population had documentation of a diagnosis of diabetes. These results suggest that a potential for increased morbidity associated with tourniquet use in civilian populations and that further research is needed to better understand the risk-benefit ratio of this intervention in this population.

The outcomes observed in this study suggest that tourniquets may be over-utilised patients who are unlikely to benefit and consequently be exposed to unnecessary harm. The majority of patients in this study did not have major trauma, with only 30% of patients meeting the criteria for major trauma according to their ISS. Median ISS was 10 (2, 16), which was similar to a systematic review which found tourniquets being applied to non-serious injuries that might have been controlled with conventional methods [9]. The majority of patients were haemodynamically stable with a shock index < 1 both prehospital and in ED. Furthermore, there were seven patients who required no surgical intervention and were able to be discharged home from the ED. These data suggests that a significant proportion of tourniquets are being applied to non-serious trauma.

Whilst our data suggests that PHTQ use is being over-utilised, it is important to acknowledge that more stringent adherence to current guidelines could result in harm in patients who may have benefited from a PHTQ, but did not meet current indications. This study along with ongoing research in the field is to ensure the development of better, evidence-based guidelines.

The work of prehospital medical staff is extremely difficult, often having to work in high acuity and time sensitive situations with limited information. The authors acknowledge the difficult work these essential workers complete and the complexity of the decision making process in these situations.

### Limitations

The present study has several limitations. The single-centre design reduces generalisability to other trauma centres although our local prehospital guidelines are followed state-wide. The retrospective nature of this study, based on the review of prehospital data sheets and inpatient medical records, makes the conclusions about decision-making related to PHTQ use limited. This could lead to some misclassification between the indicated and non-indicated categories. The classification between indicated and non-indicated by a single author is also a limitation which could have led to misclassification. Future studies should utilise independent assessment by multiple authors or a consensus panel. Patients who arrived to the hospital with already released tourniquets were excluded, some of these could have been examples of good practice, when the tourniquet were applied to control bleeding and released on reassessment when haemorrhage was arrested. Furthermore, patients with inadequate tourniquet application were not excluded from analysis as our primary aim was to assess indicated use and not adequacy of technique, which could have affected the assessment of complications. Our study also cannot cover the entire complex decision making about tourniquet use in the prehospital environment, such as situations when tourniquet was considered but based on the lack of indications not applied.

## Conclusion

This study demonstrates that the increasing incidence of PHTQ use is a result of increased non-indicated use. The majority of PHTQ use over the study period was non-indicated according to current prehospital guidelines. These patients were unlikely to benefit from the application of a PHTQ and may have been placed at risk of harm. The current guidelines should be better reinforced and greater emphasis needs to be placed on the importance of attempting direct pressure, wound packing, use of haemostatic agents and appropriate dressings before applying a tourniquet as in many instances this will negate the need. PHTQ practices warrant continuous prospective surveillance to optimise care delivery related to this lifesaving device.

## Electronic supplementary material

Below is the link to the electronic supplementary material.

Supplementary Material 1

Supplementary Material 2

## Data Availability

No datasets were generated or analysed during the current study.
