# Supplementary material for: Increasing prehospital tourniquet use attributed to non-indicated use: an 11-year retrospective study
Source: Eur J Trauma Emerg Surg. 2025 Jan 24;51(1):71. doi: 10.1007/s00068-024-02716-3 (PMC11762000; doi:10.1007/s00068-024-02716-3)
Supplement: Supplementary file 2 — Supplementary Material 2 [file 68_2024_2716_MOESM2_ESM.docx]

| **Interventions in the Emergency Department** | |
| --- | --- |
| **Intervention** | **Number of cases, n (%)** |
| Massive Transfusion Protocol | 21 (24%) |
| Blood Transfusion | 35 (43%) |
| Tranexamic acid | 20 (24%) |
| Fluids | 52 (62%) |
| Pressure bandage | 64 (76%) |
| Haemostatic bandage | 11 (13%) |
| Sedation | 14 (17%) |
| Intubation | 7 (8.5%) |
| Splinted | 4 (6.1%) |
| Venous suturing | 1 (1.2%) |
| Washout / Irrigation / closed in ED | 7 (8.5%) |

*Categorical data are presented as frequency counts and percentages.*

| **Distribution of arterial injury.** | |
| --- | --- |
| **Artery** | **Number of cases, n (%)** |
| Radial | 16 (33%) |
| Ulnar | 13 (27%) |
| Brachial | 6 (13%) |
| Popliteal | 4 (8.3%) |
| Posterior tibial | 4 (8.3%) |
| Femoral | 2 (4.2%) |
| Unnamed lower limb artery | 2 (4.2%) |
| Anterior tibial | 1 (2.1%) |

*Categorical data are presented as frequency counts and percentages.*

| **Interventions in the operating room** |  |
| --- | --- |
| **Intervention** | **Number of cases, n (%)** |
| Exploration / Debridement / Washout | 75 (87%) |
| Amputation | 9 (11%) |
| Replantation of limb | 3 (3.5%) |
| Vascular ligation (Arterial) | 8 (9.3%) |
| Vascular repair (Arterial) | 34 (40%) |
| Vascular repair (Venous) | 13 (15%) |
| Vascular ligation (Venous) | 16 (19%) |
| Vascular shunt placement | 1 (1.2%) |
| Vascular recanalization | 2 (2.3%) |
| Nerve repair | 30 (35%) |
| Tendon repair | 32 (37%) |
| Foreign body removal | 4 (4.7%) |
| Internal Fixation | 13 (15%) |
| External Fixation | 7 (8.1%) |
| Skin graft | 6 (7.1%) |
| Prophylactic fasciotomy | 3 (3.5%) |

*Categorical data are presented as frequency counts and percentages.*
